# Prediction of subclinical atherosclerosis in low Framingham risk score individuals by using the metabolic syndrome criteria and insulin sensitivity index

**DOI:** 10.3389/fnut.2022.979208

**Published:** 2022-10-24

**Authors:** Benjamin Huang, Weiting Huang, John Carson Allen, Lijuan Sun, Hui Jen Goh, Siew Ching Kong, Dewaine Lee, Cherlyn Ding, Nabil Bosco, Leonie Egli, Lucas Actis-Goretta, Faidon Magkos, Fabrizio Arigoni, Melvin Khee-Shing Leow, Swee Yaw Tan, Khung Keong Yeo

**Affiliations:** ^1^Singapore General Hospital, Singapore, Singapore; ^2^Duke-NUS Medical School, Singapore, Singapore; ^3^National Heart Center Singapore, Singapore, Singapore; ^4^Singapore Institute for Clinical Sciences, Singapore, Singapore; ^5^Nestlé Institute of Health Sciences Singapore, Singapore, Singapore; ^6^Nestlé Institute of Health Sciences, Nestlé Research, Lausanne, Switzerland; ^7^University of Copenhagen, Frederiksberg, Denmark; ^8^Department of Endocrinology, Tan Tock Seng Hospital, Singapore, Singapore; ^9^Lee Kong Chian School of Medicine, Nanyang Technological University, Singapore, Singapore

**Keywords:** subclinical atherosclerosis, metabolic syndrome and type II diabetes, insulin sensitivity, computed tomography, Framingham risk

## Abstract

**Background:**

Subclinical atherosclerosis can be present in individuals with an optimal cardiovascular risk factor profile. Traditional risk scores such as the Framingham risk score do not adequately capture risk stratification in low-risk individuals. The aim of this study was to determine if markers of metabolic syndrome and insulin resistance can better stratify low-risk individuals.

**Methods:**

A cross-sectional study of 101 healthy participants with a low Framingham risk score and no prior morbidities was performed to assess prevalence of subclinical atherosclerosis using computed tomography (CT) and ultrasound. Participants were compared between groups based on Metabolic Syndrome (MetS) and Insulin-Sensitivity Index (ISI-cal) scores.

**Results:**

Twenty three individuals (23%) had subclinical atherosclerosis with elevated CT Agatston score ≥1. Presence of both insulin resistance (ISI-cal <9.23) and fulfillment of at least one metabolic syndrome criterion denoted high risk, resulting in significantly improved AUC (0.706 95%CI 0.588–0.822) over the Framingham risk score in predicting elevated CT Agatston score ≥1, with net reclassification index of 50.9 ± 23.7%. High-risk patients by the new classification also exhibited significantly increased carotid intima thickness.

**Conclusions:**

The overlap of insulin resistance and presence of ≥1 criterion for metabolic syndrome may play an instrumental role in identifying traditionally low-risk individuals predisposed to future risk of atherosclerosis and its sequelae.

## Introduction

Estimation of subclinical disease and the future development of a cardiovascular risk profile or cardiovascular disease in the low-risk asymptomatic individual remains a challenging task. Large population studies have highlighted a high prevalence of subclinical disease across low-risk populations, thereby signaling a global need for improved future risk prognostication tools and possibly early initiation of targeted preventive measures to arrest progression of subclinical disease to clinically overt disease. This is supported by PESA (1), MESA (2, 3) and CARDIA (4) studies highlighting high prevalence of subclinical atherosclerosis verified by either ultrasound imaging or adjunct measures of coronary artery calcium score (CACS) in middle-age cohorts with extensive atherosclerosis present in traditionally classified low-risk individuals. This raises concern for future cardiovascular events.

Presently, the selection of an appropriate cardiovascular risk assessment tool is complicated by the impending dawn of future population health crises and the increasing global incidence of chronic conditions—e.g. metabolic syndrome, diabetes mellitus, atherosclerosis and chronic kidney disease. There has been significant emphasis surrounding better risk stratification of classically low-risk individuals as demonstrated by the push to further investigate the role chronic illness may play in the future global health landscape (5, 6). Clinical screening tools such as metabolic syndrome (7, 8), insulin sensitivity/resistance (9, 10), triglyceride-glucose index (11, 12) and other cardiovascular health indices (e.g. Framingham Risk score, FUSTER-BEWAT, ICHI) (6, 13) have already been suggested as robust indicators of cardiovascular disease risk in low risk populations. Additionally, there are some early promising results illustrating how potential clinical markers identified in chronic illnesses are able to improve the accuracy of the above indices in predicting future disease risk (6). Investigations involving fasting and postprandial markers such as insulin and other metabolites such as triglycerides and cholesterol have also helped to better elucidate chronic disease progression (14, 15).

Pending better understanding of subclinical disease and its relevant markers, the use of other more appropriate risk stratification systems is forthcoming. In this study we hypothesized that the use of metabolic syndrome criteria and insulin sensitivity criteria may serve as important screening tools for the presence of subclinical atherosclerosis and improve assessment of future cardiovascular disease risk in individuals with absence of traditional risk factors. A secondary aim of this study was to explore the role that mixed-meal tolerance tests (MMTT) (16, 17) may have in identifying early subclinical risk factors—e.g. post prandial profile lipid and glycemic measures—not typically assessed by traditional clinical tests.

## Methods

### Study design

The study took place at two sites: enrollment and cardiovascular assessment was performed at the National Heart Center Singapore (NHCS) and mixed-meal testing and processing of samples at the Singapore Institute for Clinical Sciences (SICS). A total of 116 healthy Chinese middle-aged participants between 40 and 54 years old were recruited from May 2018 to June 2019. Exclusion criteria included known medical conditions and respective treatment (myocardial infarction, stroke, peripheral artery disease, cancer, chronic lung diseases and any active current treatment for such), pharmacological treatment for known cardiovascular risk factors (hypertension, hyperlipidemia and diabetes mellitus), pregnancy and contraception, morbid obesity and contraindications to undergo testing (intolerance to MMTT, contraindication to cardiovascular MRI). The study was conducted in accordance with the Declaration of Helsinki and approved by the SingHealth Centralized Institutional Review Board (2018/2116. Procotol No. 17.15.NRC). Informed consent was obtained from all study participants.

### Cardiovascular assessments

Intima media thickness (IMT) is defined as a double-line pattern visualized by ultrasound on both walls of the carotid vessel. CT Agatston score for Coronary Artery Calcification (CAC) was determined by means of a non-contrast CT scan. Presence of subclinical atherosclerosis was defined as the presence of at least one plaque or a CAC ≥1 (18). Abnormal carotid intima thickness was defined as IMT more than 0.9 mm while ultrasound-guided criteria for subclinical atherosclerosis with focal wall thickening >50% of the surrounding vessel wall or IMT >1.5 mm (19).

### Mixed meal tolerance test

Blood sampling was performed to quantify blood markers after 10 hours of imposed fasting and then at frequent intervals over the duration of the mixed-meal test. Insulin, glucose and C-peptide were measured at 0, 10, 20, 30, 45, 60, 90, 120, and 240 minutes, while other markers including lipids and triglycerides were measured at 0, 60, 120, and 240 minutes. The meal challenge consisted of 237.5 mL of a nutritional drink (Nestle Heath Sciences, Lausanne, Switzerland) mixed with 100 mL of commercially available whipping cream. This composition was determined based on a systematic review that defined an optimal nutritional stress test (20): 75 g glucose, 60 g palm olein and 20 g dairy protein served in a ~337 mL liquid meal providing a total of ~930 kcal.

### Definitions

Framingham risk and Metabolic Syndrome (MetS) criteria were defined according to established guidelines by the Framingham Heart Study and International Diabetes Federation respectively (21, 22). We used the Framingham risk score formula adapted for the Singaporean population (23) to determine that the study sample aligned with the criteria of low Framingham risk individuals. Predicted Insulin sensitivity index (ISI-cal) was defined using a local cohort study and calculated by ISI-cal = 14.15 ^*^ (waist-to-hip ratio [WHR])^−2.63^^*^ (fasting insulin [I_0]_)^0.39^^*^ (triglycerides[TG])^0.21^ with a threshold of ≤ 9.23 for increased future risk of cardiovascular disease (14). Plasma insulinogenic response was calculated by the ratio of insulin/glucose area-under-the-curve (AUC) (24).

The sample was segmented into four distinct risk groups based on the following criteria: low risk (no MetS criteria, ISI-cal normal), intermediate risk Mod-1 (ISI-cal abnormal, no MetS criteria), intermediate risk Mod 2 (at least 1 MetS criteria, ISI-cal normal), high risk (at least 1 MetS criteria, ISI-cal abnormal).

### Statistical analysis

Statistical comparisons were performed between participants sorted according to presence or absence of ≥1 MetS Criteria and ISI-cal scoring ≤ 9.23. Fisher's exact test was used to analyze categorical variables, while Student's *t*-tests and Wilcoxon tests were used to analyze continuous variables depending on normality of the data. Analysis of variance (ANOVA) was used to analyze multiple group comparisons. Receiver operator characteristics (ROC) curve analysis was used to assess accuracy of the different scoring systems in identifying subclinical atherosclerosis.

Estimation of biomarker response was tabulated by fasting, maximum and area-under-curve (AUC). Data used to compute these variables for comparisons included samples obtained at time points previously stated during the MMTT. The AUC was calculated for parameters of pancreatic beta-cell function using the trapezoidal method. Statistical analyses were performed with SAS (SAS ^®^, Cary, NC, USA) PROC MIXED, Version 9.3. Models were adjusted for age and gender. Statistical significance was defined as two-sided *p*-value < 0.05 unless otherwise stated.

## Results

A total of 101 (46 females, 55 males) out of 116 healthy Chinese middle-aged participants between 40 and 54 years old were included in this study, after applying exclusion criteria and completion of all study visits. Participants were sorted according to presence of ≥1 MetS criteria and meeting criteria for ISI-cal insulin sensitivity index for future risk of cardiovascular disease. There were no significant differences in age and gender across all categories (Table 1).

**Table 1:** Baseline characteristics of groups with and without concurrence of metabolic syndrome criteria (MetS) and predicted insulin sensitivity index (ISI-cal).

**Variable**	**Mean**				***p*-value**	**Inter-group comparisons**					
	**Low risk^a^**	**Mod-1^b^**	**Mod-2^c^**	**High risk^d^**		**Low vs. High**	**Low vs. Mod-1**	**Low vs Mod-2**	**High vs. Mod-1**	**High vs. Mod-2**	**Mod-1 vs. Mod-2**
	**(*N* = 41)**	**(*N* = 13)**	**(*N* = 15)**	**(*N* = 32)**							
**Clinical Characteristics**
Age, Years	48.3 ± 4.3	47.2 ± 4.4	47.9 ± 5.2	46.9 ± 3.7	0.541	0.167	0.385	0.714	0.878	0.489	0.661
Male Gender (%)	21 (51)	4 (31)	10 (67)	20 (63)	0.193	0.354	0.222	0.372	0.098	1.000	0.128
Height, m	1.66 ± 0.08	1.63 ± 0.08	1.67 ± 0.08	1.68 ± 0.08	0.382	0.456	0.216	0.815	0.085	0.736	0.220
Weight, kg	59.8 ± 8.2	64.3 ± 6.7	63.6 ± 8.1	73.4 ± 10.1	<.0001	<.0001	0.113	0.150	0.002	0.001	0.851
BMI, kg/m^2^	21.59 ± 1.86	24.15 ± 1.63	22.83 ± 2.35	26.03 ± 2.44	<.0001	<.0001	0.000	0.059	0.009	<.0001	0.108
Systolic Blood Pressure, mmHg	109 ± 11	113 ± 11	113 ± 9	120 ± 14	0.003	0.000	0.233	0.289	0.117	0.067	0.874
Diastolic Blood Pressure, mmHg	73 ± 9	77 ± 6	76 ± 7	84 ± 11	<.0001	<.0001	0.197	0.244	0.019	0.008	0.875
Waist Circumference, cm	75.3 ± 6.5	83.9 ± 5.1	81.4 ± 5.3	90.3 ± 8.3	<.0001	<.0001	0.000	0.004	0.005	<.0001	0.330
Waist Hip Ratio	0.87 ± 0.05	0.87 ± 0.03	0.92 ± 0.03	0.93 ± 0.04	<.0001	<.0001	0.267	<.0001	<.0001	0.219	0.004
Smoker (%)	5 (12)	1 (8)	1 (7)	3 (9)	1.000	1.000	1.000	1.000	1.000	1.000	1.000
Metabolic Syndrome Score	–	1.08 ± 0.00	–	1.41 ± 0.56	–	–	–	–	–	–	–
**Cardiovascular assessment characteristics**
CT Agatston score	0.00 (0.00)	0.00 (4.00)	0.00 (0.50)	0.00 (6.00)	0.081	0.088	0.131	0.499	1.000	0.899	0.857
Ln (1 + CT Agatston score)	0.00 (0.00)	0.00 (1.61)	0.00 (0.35)	0.00 (1.95)	0.081	0.088	0.131	0.499	1.000	0.899	0.857
cIMT, mm	0.52 ±	0.56 ±	0.53 ±	0.56 ±	0.135	0.035	0.107	0.608	0.965	0.268	0.341
**Fasting characteristics**
Glucose, mmol/l	5.27 ± 0.42	5.43 ± 0.40	5.43 ± 0.58	5.50 ± 0.45	0.196	0.038	0.278	0.256	0.649	0.627	0.995
Insulin, μU/ml	5.16 ± 2.14	5.35 ± 1.40	8.96 ± 3.34	12.69 ± 6.37	<.0001	<.0001	0.882	0.002	<.0001	0.004	0.020
Cpeptide, ng/ml	1.50 ± 0.42	1.60 ± 0.36	2.16 ± 0.62	2.71 ± 0.86	<.0001	<.0001	0.596	0.001	<.0001	0.005	0.017
Total Cholesterol, mmol/l	5.05 ± 0.70	5.00 ± 0.69	5.46 ± 0.76	5.23 ± 0.87	0.266	0.318	0.834	0.079	0.358	0.341	0.115
LDL, mmol/l	3.08 ± 0.59	3.11 ± 0.65	3.57 ± 0.58	3.33 ± 0.80	0.069	0.111	0.879	0.015	0.316	0.242	0.068
HDL, mmol/l	2.04 ± 0.34	1.95 ± 0.41	1.65 ± 0.44	1.60 ± 0.41	<.0001	<.0001	0.477	0.001	0.008	0.712	0.042
Total TG, mmol/l	0.76 ± 0.25	0.81 ± 0.34	1.31 ± 0.63	1.48 ± 0.62	<.0001	<.0001	0.699	0.000	<.0001	0.250	0.007

a Low risk: MetS and ISI-cal criteria not fulfilled.

b Mod-1 (Intermediate risk): ≥1 MetS criteria and ISI-cal criteria >9.23.

c Mod-2 (Intermediate risk): ISI-cal criteria ≤ 9.23 and MetS 0.

d High risk: MetS and ISI-cal criteria both fulfilled.

Of note, there was decent concurrence of categorization between both criteria with 73/101 (73%) overlap. Forty-one participants did not fulfill MetS and ISI-cal criteria while 32 participants met both criteria. The remaining 27 participants were separated into groups of 13 and 15 individuals who met only ≥1 MetS criteria or ISI-cal criteria ≤ 9.23 respectively. Forty-five participants fulfilled partial ≥1 MetS criteria, while only 1 participant met MetS criteria (at least 3 of 5 criteria). Forty-seven participants met ISI-cal criterion of ≤ 9.23.

The metabolic syndrome criteria met in this cohort included waist circumference [28 (62%)] blood pressure [9 (20%)], fasting triglycerides [14 (31%)], fasting high density cholesterol [7 (16%)] and fasting blood glucose [1 (1%)]. Only 1 participant met thresholds for impaired fasting glucose, and no participants met thresholds for diabetes mellitus respectively (Supplementary Table 1).

### Cardiovascular assessment association with metrics of metabolic syndrome, insulin sensitivity and Framingham risk score

Twenty three (23%) participants were found to have a positive CAC score. There were no patients with abnormal IMT of more than 0.9mm. The Singapore-adapted Framingham risk score was significantly correlated with the natural logarithm of CAC (*p* = 0.019) and presence of CAC ≥1 (*p* = 0.033); and it only tended to be correlated with carotid IMT (*p* = 0.099).

When participants were categorized by the absence or presence of ≥1 MetS criteria, CAC (its natural logarithm: 1 + CAC) and cIMT were found to be significantly different between groups (Supplementary Table 1). Categorization based on ISI-cal scoring revealed no statistical significance for CAC, its natural logarithm and cIMT (Supplementary Table 2).

### Interaction of partial fulfillment of MetS criteria and insulin sensitivity

We categorized patients into 4 new risk groups: low risk (no MetS criteria, ISI-cal normal), Mod-1 (at least 1 MetS criteria, ISI-cal normal), Mod 2 (ISI-cal abnormal, no MetS criteria), high risk (at least 1 MetS criteria, ISI-cal abnormal); there were no significant differences in CT Agatston score (*p*-value for trend = 0.081; Table 1) and Ln (1 + CAC) (*p*-value for trend= 0.081).

When compared to the low risk group, the odds ratio of the high risk group having CAC ≥1 was 3.58 (95%CI 1.06–12.09, *p* = 0.039), even after adjusting for the Framingham risk score. Although no participant had a significantly elevated carotid IMT of more than 0.9 mm, higher “new” risk remained significantly associated with a thicker IMT (*p* = 0.045) after adjusting for the Framingham Risk Score.

### Receiver operating characteristic curve analysis on utility of scores in predicting subclinical atherosclerosis

**Figure 1:**
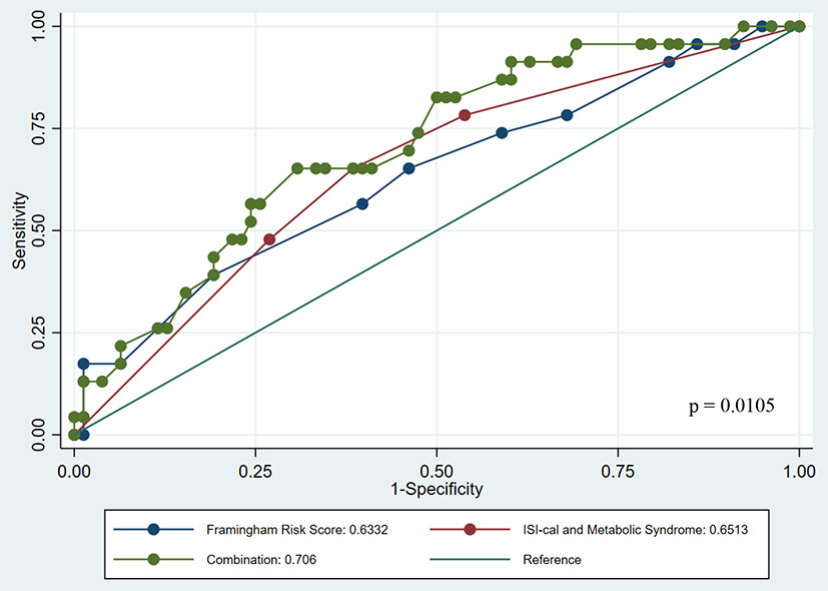
Receiver Operator Curve comparing role of risk stratification scores across Framingham risk, ISI-cal and Metabolic Syndrome separately and combined.

Examining the predictive ability of this new grouping, we compared the area under the curve (AUC) for: Framingham risk score (AUC 0.633, 95%CI 0.499–0.766), new risk groups (AUC 0.651, 95%CI 0.530–0.771) and a combination of both Framingham risk score and new risk groups (AUC 0.706, 95%CI 0.588–0.822; Figure 1). The combination was significantly better than Framingham risk score and new risk groups when they were used alone (*p* = 0.010). The net reclassification index of using both the Framingham risk score and new risk groups over Framingham risk score alone was 50.9 ± 23.7%, *p*-value = 0.0318, and the integrated discriminant improvement was 4.66 ± 2.40%, p-value = 0.050.

We tested the interaction of the new risk groups with individual components of MetS, and it was significant for elevated blood pressure (*p* = 0.017) and low high density lipoprotein cholesterol (*p* = 0.047), suggesting additional risk modification when these 2 factors are present. Of note the number of MetS components present was not significant when tested for interaction with the new risk groups (*p* = 0.238).

### Inter-group variation of fasting and beta-cell function measures from MMTT

Groups classified into the 4 new risk groups were significantly different in fasting measures of insulin, C-peptide, HDL cholesterol and triglyceride (Table 1). Fasting insulin levels were notably higher across the intermediate risk group determined by ISI-cal and the high risk group determined by fulfillment of MetS and ISI-cal when compared to low risk groups (Table 1). Plasma insulinogenic responses (AUC of Insulin:Glucose) were significantly elevated across the intermediate risk group determined by ISI-cal and the high risk group and were about 1.5 to two fold greater than in low risk groups (Supplementary Table 3). Postprandial maximal values for glucose, insulin, C-peptide, triglycerides and HDL cholesterol were significantly different across the 4 new risk groups (Supplementary Table 3).

## Discussion

Unlike previous larger scale studies that suggested a high rate of subclinical atherosclerosis in middle aged individuals, our results highlighted a lower prevalence of 20–30% subclinical atherosclerosis (CAC ≥1) in low Framingham risk individuals (<10%) without traditional risk factors. Notably, our cohort did not have plaques meeting ultrasound-guided criteria for subclinical atherosclerosis and arterial injury (i.e. with focal wall thickening >50% of the surrounding vessel wall or IMT >1.5mm), thereby suggesting low severity of subclinical disease. This was further supported by a large proportion of individuals with CAC ≥1 registering mild scores between 0 and 99 (*n* = 19; 83%), while further stratification of participants revealed that 10 (43%) had scores 1–20. These results are likely robust alongside the literature suggesting that severity of MetS and influence of cardiovascular factors such as diabetes is correlated with frequency and severity of atherosclerotic disease burden (25). These results potentially highlight that the absence of traditional risks likely yields a fairly low prevalence of subclinical atherosclerosis as compared to previous studies that may have not differentiated groups based on cardiovascular risk status.

Amongst individuals with subclinical disease and early insulin resistance, CAC, ln (1+CAC) and cIMT were different between groups categorized by absence or presence of ≥1 MetS (Supplementary Table 1) while none of the cardiovascular assessments were different when groups were categorized by ISI-cal criteria (Supplementary Table 2). Nevertheless, our ROC analyses demonstrated that the use of the ISI-cal index alongside MetS proved comparable with Framingham risk score (Figure 1). We recognize that the non-difference in inter-group comparisons potentially suggests ISI-cal may not be a robust enough standalone tool in differentiating individuals with milder forms of coronary calcification (CACS 1–20), but its utility may still be warranted in view of it being more sensitive for predicting future cardiovascular disease and events (14). As such, the combination of partial fulfillment of MetS and prevalence of insulin resistance may help identify the presence of subclinical atherosclerosis while also predicting future risk (8). Further studies investigating appropriate cut offs of elevated CAC between more clearly defined risk groups may assist to better explain how we may better interpret the use of such objective testing in the clinical setting (26).

In addition, the results from this study and a search of the literature suggest that the use of other markers such as fasting insulin, triglycerides and waist-hip ratio as illustrated by the ISI-cal index are in keeping with identifying asymptomatic individuals at risk of cardiovascular disease (14, 27). It has been well established that early dysregulation in glycemic measures and insulin resistance predispose individuals to future cardiovascular disease (28), but studies looking at the long term risks in low Framingham risk groups are still lacking. Population studies have revealed that individuals with non-major cardiovascular diseases may be better screened for future cardiovascular disease by other well established screening tools such as MetS (8, 25, 29) and there is a growing evidence for other insulin resistance models to explain presence of subclinical and future cardiovascular risk (27, 30–32). A supporting study by Mostaza et al. showed that glycemic control may be independently associated with all grades of atherosclerosis (33) which suggests the importance of stratifying individuals based on glycemic status and insulin resistance.

Despite the lower prevalence in our study compared to other larger studies, we believe what potentially supports the utility of screening individuals early is the subclinical progression of disease. A recent review demonstrated progression of plaques potentially occurred over an average course of 2–3 years prior to acute events (34). This suggests the importance of counseling individuals at risk concerning the possibility of plaque formation and recommending intervention with including lipid lowering therapy. This is further supported by the rather quick (3 years) progression of subclinical atherosclerosis as established by findings of the PESA (35). As such, our study suggests that identifying individuals at risk of subclinical disease presents an opportunity for providing early risk management advice for any needed interventions.

A secondary aim of this study was to explore the potential utility the MMTT may have in individuals presenting with absence of traditional cardiovascular risk factors (16, 17). It is important to note that only one participant meeting criteria for impaired fasting glucose and no participants meeting criteria for diabetes mellitus. In this setting, the utility of 4 hour MMTT data was considered for future clinical testing (36). While absence of non-insulin dependent diabetes mellitus in this cohort could be verified by traditional glucose testing, higher basal fasting insulin >10 uIU/ml, higher 4-hour insulin AUC ~16,000-17,000 uIU/ml min and two-fold increases in plasma insulin: glucose ratio suggested there is some notable degree of insulin resistance within participants with incomplete metabolic syndrome criteria or meeting criteria for ISI-cal scoring in this cohort (37, 38).

Mechanisms for early development of atherosclerosis were also supported by MMTT. Postprandial dyslipidemia has been previously suggested to play a significant role early in the formation of atherosclerosis through the imbalance of vasodilatory and vasoconstrictive responses leading to endothelial dysfunction and oxidative stress (15). This may be important considering that individuals are more often in a non-fasting state, spending most time in the postprandial state. This phenomenon was further supported by significant differences in postprandial dyslipidemia with higher levels identified in high risk groups categorized by incomplete Metabolic Syndrome and/or meeting criteria as per ISI-cal index (Supplementary Table 3). Participants in this group had postprandial triglyceridemia >2.27 and >2.5 mmol/L (15, 39) beyond minimum and desirable thresholds suggested in the literature.

The literature also suggests that the rates of endothelial dysfunction could be stratified by presence of family history in early pre-diabetic individuals compared to normal glucose tolerance individuals. Endothelial dysfunction was more pronounced in spite of similar post prandial triglyceride levels between pre-diabetics without family history and with normal glucose tolerance individuals (40). Our findings suggest that stratification of individuals by clinical tools and guidelines may play important roles in recommending early intervention and preventing worsening subclinical cardiovascular disease by conservative measures such as dietary advice and exercise.

## Strengths and limitations

This study potentially positions itself as being clinically useful as it is seeks to uncover the risk of subclinical atherosclerosis in a population subgroup typically deemed to be at reduced risk of developing future cardiovascular disease. The middle age group studied is appropriately at the juncture where cardiovascular risk is typically projected to be in its early stages, while the utility of the clinical markers used to further categorize risk groups is supported by various population studies. Of note, this is also possibly one of the first studies illustrating the use of postprandial markers in re-stratifying long term cardiovascular risk in an Asian population.

The authors note that the study may be weakly powered in view of the small sample size (*n* = 101) and presence of residual bias from factors not measured. The authors also recognize that the literature currently suggests there may be clinical limitations in the role of assessing subclinical atherosclerosis and its use in prognosticating risk. Non-etheless, given the established use of clinical tools in prognosticating long term risk, it may suggest that these tools can serve multiple clinically useful functions. Hence, it is encouraging that the findings described have highlighted how we may be better able to identify individuals at risk of subclinical atherosclerosis. Further larger scale studies exploring the utility of clinical tools and postprandial testing in identifying typically low risk individuals with subclinical atherosclerosis will be required to further justify its clinical utility.

## Conclusions

Utilizing Metabolic Syndrome and Insulin Sensitivity criteria as clinical screening tools in non-diabetic individuals with low Framingham risk scores may help refine future cardiovascular disease risk stratification.

## Data availability statement

The original contributions presented in the study are included in the article/Supplementary material, further inquiries can be directed to the corresponding author/s.

## Ethics statement

The studies involving human participants were reviewed and approved by SingHealth Centralized Institutional Review Board (2018/2116. Procotol No. 17.15.NRC). The patients/participants provided their written informed consent to participate in this study.

## Author contributions

BH, WH, KY, ML, FA, NB, and FM designed the study and helped with the interpretation of data, discussion, and reviewing of the manuscript. BH, WH, and JA designed and performed statistical analysis. BH and WH analyzed data. BH, WH, and KY wrote the paper and had primary responsibility for final content. KY, ML, SK, and LS had primary responsibility for the good conduct of the clinical trial. All authors have read and approved the final manuscript.

## Funding

This work was supported financially by the Agency for Science, Technology and Research (A^*^STAR), Singapore (Grant No. I-1701E0B15), Lee foundation through support of the SingHeart study, and core funding from SingHealth and Duke NUS through their institute of Precision Medicine (PRISM) and a center grant awarded to National Heart Centre Singapore from the National Medical Research Council, Ministry of Health, Republic of Singapore (Grant No. NMRC/CG/M006/2017_NHCS). The funding bodies were not involved in design, conduct, data interpretation, or writing of the manuscript.

## Conflict of interest

Author KY has received research funding from Medtronic and honoraria from Abbott Vascular, Boston Scientific, Amgen, and Menarini. Authors KY, FM, and ML have received funding from their respective academic institutions and Nestlé to conduct the study. At the time of study, authors LE, NB, FA, and LA-G were Nestlé employees. FM is a member of the editorial board of AJCN. The remaining authors declare that the research was conducted in the absence of any commercial or financial relationships that could be construed as a potential conflict of interest.

## Publisher's note

All claims expressed in this article are solely those of the authors and do not necessarily represent those of their affiliated organizations, or those of the publisher, the editors and the reviewers. Any product that may be evaluated in this article, or claim that may be made by its manufacturer, is not guaranteed or endorsed by the publisher.

## Abbreviations

AUC: Area-under-curve
CACS: Coronary Artery Calcium score
cIMT: Carotid Intima-Media Thickness
HDL: High Density Lipoprotein
ISI-cal: Predicted Insulin Sensitivity Index
MetS: Metabolic Syndrome
MMTT: Mixed Meal Tolerance Test
TG: Triglyceride.
