# Supplementary material for: Prediction of subclinical atherosclerosis in low Framingham risk score individuals by using the metabolic syndrome criteria and insulin sensitivity index
Source: Front Nutr. 2022 Oct 24;9:979208. doi: 10.3389/fnut.2022.979208 (PMC9639788; doi:10.3389/fnut.2022.979208)
Supplement: Supplementary file 1 [file Data_Sheet_1.docx]

**Appendix**

**Supplementary Table 1. Cohort Descriptive Statistics categorized by Presence of ≥1 Metabolic Syndrome Criteria**

| **Variable** | **Mean** | | **Difference** | **95% LCL** | **95% UCL** | **P-value** |
| --- | --- | --- | --- | --- | --- | --- |
|  | **MetS 0** | **MetS 1-5** |  |  |  |  |
|  | **(N=56)** | **(N=45)** |  |  |  |  |
| **Clinical Characteristics** | | | | | | |
| Age, Years | 48.2 ± 4.5 | 47.0 ± 3.9 | 1.2 | -0.5 | 2.9 | 0.155 |
| Male Gender (%) | 31 (55) | 24 (53) | - | - | - | 0.844 |
| Height, m | 1.66 ± 0.08 | 1.66 ± 0.08 | 0.00 | -0.03 | 0.03 | 0.968 |
| Weight, kg | 60.8 ± 8.3 | 70.8 ± 10.3 | -10.0 | -13.6 | -6.3 | <.0001 |
| BMI, kg/m^2^ | 21.92 ± 2.04 | 25.49 ± 2.47 | -3.56 | -4.46 | -2.68 | <.0001 |
| Systolic Blood Pressure, mmHg | 110 ± 10 | 118 ± 14 | -8 | -13 | -3 | 0.002 |
| Diastolic Blood Pressure, mmHg | 74 ± 9 | 82 ± 10 | -8 | -12 | -4 | <.0001 |
| Waist Circumference, cm | 77.0 ± 6.7 | 88.5 ± 8.0 | -11.5 | -14.4 | -8.6 | <.0001 |
| Waist Hip Ratio | 0.87 ± 0.06 | 0.92 ± 0.05 | -0.05 | -0.07 | -0.02 | <.0001 |
| Smoker (%) | 6 (11) | 4 (9) | - | - | - | 1.00 |
| Metabolic Syndrome Criteria (1/2/3)* | - | 32/12/1 | - | - | - | - |
| ISI-cal score | 11.44 ± 3.63 | 7.83 ± 2.74 | 3.91 | 2.64 | 5.17 | <.0001 |
| Framingham Risk Score | 7.04 ± 2.05 | 6.56 ± 3.71 | 0.48 | -0.82 | 1.78 | 0.464 |
| **Cardiovascular Assessment Characteristics** | | | | | | |
| CT Agatston score | 0.00 (0.00) | 0.00 (5.00) | - | - | - | 0.021 |
| Ln (1+CT Agatston score) | 0.00 (0.00) | 0.00 (1.78) | - | - | - | 0.021 |
| cIMT, mm | 0.52 ± 0.08 | 0.56 ± 0.09 | -0.04 | -0.07 | -0.01 | 0.021 |
| **Fasting Characteristics** | | | | | | |
| Glucose, mmol/l | 5.31 ± 0.47 | 5.48 ± 0.44 | -0.18 | -0.36 | 0.00 | 0.073 |
| Insulin, µU/ml | 6.18 ± 2.87 | 10.57 ± 6.34 | -4.43 | -6.35 | -2.51 | <.0001 |
| Cpeptide, ng/ml | 1.68 ± 0.55 | 2.39 ± 0.91 | -0.72 | -1.02 | -0.43 | 0.0004 |
| Total Cholesterol, mmol/l | 5.13 ± 0.73 | 5.16 ± 0.82 | -0.06 | -0.37 | 0.24 | 0.979 |
| LDL, mmol/l | 3.21 ± 0.61 | 3.27 ± 0.76 | -0.11 | -0.37 | 0.15 | 0.685 |
| HDL, mmol/l | 1.93 ± 0.41 | 1.70 ± 0.44 | 0.24 | 0.08 | 0.40 | 0.007 |
| Total TG, mmol/l | 0.90 ± 0.46 | 1.29 ± 0.63 | -0.42 | -0.63 | -0.21 | 0.001 |

**MetS: Metabolic Syndrome criteria. No participants had MetS score >3
- MetS 0: Absence of criteria
- MetS 1-5: Partial to fulfillment of criteria of MetS**

**Supplementary Table 2. Cohort Descriptive Statistics categorized by Insulin Sensitivity Index Threshold (ISI-cal)**

| **Variable** | **Mean** | | **Difference** | **95% LCL** | **95% UCL** | **P-value** |
| --- | --- | --- | --- | --- | --- | --- |
|  | **ISI-cal >9.23** | **ISI-cal ≤9.23** |  |  |  |  |
|  | **(N=54)** | **(N=47)** |  |  |  |  |
| **Clinical Characteristics** | | | | | | |
| Age, Years | 48.1 ± 4.3 | 47.2 ± 4.2 | 0.9 | -0.87 | 2.51 | 0.336 |
| Male Gender (%) | 25 (46) | 30 (64) | - | - | - | 0.109 |
| Height, m | 1.66 ± 0.08 | 1.67 ± 0.08 | -0.01 | -0.05 | 0.01 | 0.235 |
| Weight, kg | 60.9 ± 7.9 | 70.3 ± 10.8 | -9.4 | -13.1 | -5.72 | <.0001 |
| BMI, kg/m^2^ | 22.20 ± 2.18 | 25.91 ± 2.82 | -2.80 | -3.79 | -1.82 | <.0001 |
| Systolic Blood Pressure, mmHg | 110 ± 11 | 117 ± 13 | -7 | -12 | -3 | 0.003 |
| Diastolic Blood Pressure, mmHg | 74 ± 9 | 81 ± 10 | -7 | -11 | -4 | 0.0001 |
| Waist Circumference, cm | 77.4 ± 7.1 | 87.5 ± 8.5 | -10.1 | -13.2 | -7.0 | <.0001 |
| Waist Hip Ratio | 0.86 ± 0.05 | 0.93 ± 0.04 | -0.07 | -0.09 | -0.06 | <.0001 |
| Smoker (%) | 6 (11) | 4 (9) | - | - | - | 0.748 |
| Metabolic Syndrome Score >1 | 19 (36) | 19 (41) | - | - | - | 0.780 |
| ISI-cal score | 12.56 ± 2.83 | 6.70 ± 1.42 | 5.86 | 4.95 | 6.76 | <.0001 |
| Framingham Risk Score | 6.63 ± 3.04 | 7.04 ± 3.51 | -0.41 | -1.70 | 0.87 | 0.527 |
| **Cardiovascular Assessment Characteristics** | | | | | | |
| CT Agatston score | 0.00 (0.00, 0.00) | 0.00 (0.00, 1.00) | - | - | - | 0.132 |
| Ln (1 + CT Agatston score) | 0.00 (0.00, 0.00) | 0..00 (0.00, 0.69) | - | - | - | 0.132 |
| cIMT, mm | 0.53 ± 0.08 | 0.55 ± 0.08 | -0.02 | -0.05 | 0.01 | 0.186 |
| **Fasting Characteristics** | | | | | | |
| Glucose, mmol/l | 5.31 ± 0.41 | 5.47 ± 0.50 | -0.14 | -0.32 | 0.04 | 0.116 |
| Insulin, µU/ml | 5.21 ± 1.87 | 11.50 ± 5.75 | -5.72 | -7.44 | -4.00 | <.0001 |
| Cpeptide, ng/ml | 1.52 ± 0.37 | 2.54 ± 0.84 | -0.96 | -1.21 | -0.70 | <.0001 |
| Total Cholesterol, mmol/l | 5.04 ± 0.69 | 5.31 ± 0.84 | -0.23 | -0.53 | 0.07 | 0.136 |
| LDL, mmol/l | 3.08 ± 0.58 | 3.41 ± 0.75 | -0.29 | -0.56 | -0.03 | 0.060 |
| HDL, mmol/l | 2.02 ± 0.37 | 1.62 ± 0.41 | 0.41 | 0.25 | 0.56 | <.0001 |
| Total TG, mmol/l | 0.77 ± 0.26 | 1.42 ± 0.63 | -0.64 | -0.83 | -0.45 | <.0001 |

**ISI-cal: Predicted Insulin Sensitivity Index
- ISI-cal >9.23: Absence of ISI-cal criteria
- ISI-cal ≤9.23: Fulfillment of ISI-cal criteria**

**Supplementary Table 3. Mixed Meal Tolerance Test Analysis of variance across groups with and without concurrence of Metabolic Syndrome Criteria and Insulin Sensitivity Index Threshold (ISI-cal)**

| **Variable** | **Mean** | | | | **P-value** | **Inter-group comparisons** | | | | | |
| --- | --- | --- | --- | --- | --- | --- | --- | --- | --- | --- | --- |
|  | **Low Risk^a^** | **Mod-1^b^** | **Mod-2^c^** | **High Risk^d^** |  | **Low vs High** | **Low vs Mod-1** | **Low vs Mod-2** | **High vs Mod-1** | **High vs Mod-2** | **Mod-1 vs Mod-2** |
|  | **(N=41)** | **(N=13)** | **(N=15)** | **(N=32)** |  |  |  |  |  |  |  |
| **Metabolomic Characteristics** | | | | | | | | | | | |
| Max Glucose, mmol/l | 6.84 ± 0.99 | 7.10 ± 0.78 | 7.31 ± 0.29 | 7.58 ± 1.41 | 0.048 | 0.006 | 0.459 | 0.169 | 0.197 | 0.434 | 0.633 |
| AUC Glucose | 1378 ± 186 | 1450 ± 3102 | 1479 ±50 | 1505 ± 210 | 0.046 | 0.007 | 0.250 | 0.089 | 0.395 | 0.675 | 0.694 |
| Max Insulin, µU/ml | 64.37 ± 39.21 | 70.64 ± 28.65 | 100.04 ± 17.60 | 134.83 ± 103.34 | 0.000 | <.0001 | 0.773 | 0.086 | 0.005 | 0.106 | 0.258 |
| AUC Insulin | 8193 ± 3482 | 10292 ± 3102 | 14423 ± 1243 | 19065 ± 13197.92 | 0.004 | <.0001 | 0.442 | 0.001 | 0.001 | <.0001 | 0.125 |
| AUC Insulin:Glucose | 5.94 ± 2.27 | 7.13 ± 2.12 | 9.58 ± 1.46 | 12.74 ± 8.9 | <.0001 | <.0001 | 0.511 | 0.036 | 0.003 | 0.077 | 0.255 |
| Max Cpeptide, ng/ml | 6.77 ± 1.96 | 7.82 ± 2.14 | 9.51 ± 0.86 | 11.31 ± 0.59 | <.0001 | <.0001 | 0.327 | 0.007 | 0.002 | 0.086 | 0.181 |
| AUC Cpeptide | 1184 ± 314 | 1401 ± 273 | 1701 ± 129 | 1973 ± 89 | <.0001 | <.0001 | 0.179 | 0.001 | 0.001 | 0.086 | 0.117 |
| Max Total Cholesterol, mmol/l | 5.26 ± 0.77 | 5.25 ± 0.74 | 5.65 ± 0.87 | 5.37 ± 0.89 | 0.441 | 0.549 | 0.974 | 0.119 | 0.645 | 0.289 | 0.203 |
| AUC Total Cholesterol | 1224 ± 181 | 1224 ± 171 | 1312 ± 195 | 1260 ± 217 | 0.470 | 0.437 | 0.998 | 0.139 | 0.575 | 0.401 | 0.237 |
| Max LDL, mmol/l | 3.16 ± 0.62 | 3.19 ± 0.65 | 3.63 ± 0.61 | 3.35 ± 0.79 | 0.136 | 0.239 | 0.890 | 0.025 | 0.476 | 0.198 | 0.094 |
| AUC LDL | 726 ± 143 | 729 ± 150 | 828 ± 149 | 765 ± 192 | 0.190 | 0.309 | 0.952 | 0.039 | 0.497 | 0.219 | 0.108 |
| Max HDL, mmol/l | 2.10 ± 0.36 | 2.02 ± 0.42 | 1.67 ± 0.46 | 1.63 ± 0.43 | <.0001 | <.0001 | 0.508 | 0.001 | 0.004 | 0.718 | 0.028 |
| AUC HDL | 477 ± 80 | 461 ± 99 | 382 ± 109 | 368 ± 99 | <.0001 | <.0001 | 0.593 | 0.001 | 0.003 | 0.629 | 0.029 |
| Max Total TG, mmol/l | 1.82 ± 0.65 | 2.11 ± 0.75 | 3.10 ± 1.14 | 3.44 ± 1.61 | <.0001 | <.0001 | 0.439 | 0.000 | 0.001 | 0.356 | 0.027 |
| AUC Total TG | 332 ± 104 | 360 ± 124 | 539 ± 192 | 615 ± 229 | <.0001 | <.0001 | 0.614 | 0.000 | <.0001 | 0.162 | 0.008 |

**^a^Low risk: MetS and ISI-cal criteria not fulfilled ^b^Mod-1 (Intermediate risk): ≥1 MetS criteria and ISI-cal criteria >9.23
^c^Mod-2 (Intermediate risk): ISI-cal criteria ≤9.23 and MetS 0
^d^High risk: MetS and ISI-cal criteria both fulfilled**
